# HPLC-DAD technique for the quantification of a recently approved anti-diabetic triple combination along with two toxic official impurities: Toxicity confirmation aided by molecular docking application

**DOI:** 10.1186/s13065-023-00927-0

**Published:** 2023-03-15

**Authors:** Eman A. Bahgat, Hisham Hashem, Hanaa Saleh, Ebraam B. Kamel, Maya S. Eissa

**Affiliations:** 1 Pharmaceutical Analytical Chemistry Department, Faculty of Pharmacy, Zagazig University, Zagazig, 44519 Egypt; 2 Pharmaceutical Chemistry Department, Faculty of Pharmacy, Egyptian Russian University, Badr City, Cairo, 11829 Egypt

**Keywords:** Empagliflozin, Linagliptin, Molecular docking, Toxic impurities

## Abstract

**Background:**

Gliflozins and gliptins are two distinct groups of pharmacological drugs that reduce blood glucose levels in individuals with type II diabetes in various ways that may perform their functions harmoniously. Trijardy^®^ tablet, which contains empagliflozin, linagliptin, and metformin, was recently approved. The scientific database does not yet have a method that is sensitive enough to quantify the aforementioned medications in the presence of metformin official toxic impurities melamine and cyanoguanidine. Molecular docking modeling was utilized in this work to further prove the toxicity of melamine.

**Methods:**

The five analytes listed before were quantified using RP-HPLC-diode array detector and a Zorbax^®^ C8 column (4.6 × 250 mm, 5 μm) with isocratic mobile phase composed of acetonitrile and 0.05 M potassium dihydrogen phosphate buffer, which had been treated by ?-phosphoric acid to restore a pH of 4.0 (90:10, v/v) at a flow rate of 1.2 mL/min and the eluted peaks were scanned at 250 nm.

**Conclusion:**

The utilization of the simplest isocratic elution mode give the current technique a significant time-and cost-saving benefit. The current method can quantify the triple therapy agents in the presence of each other as well as with two official toxic impurities of metformin in one short analytical run.

**Supplementary Information:**

The online version contains supplementary material available at 10.1186/s13065-023-00927-0.

## Introduction

**Fig. 1:**
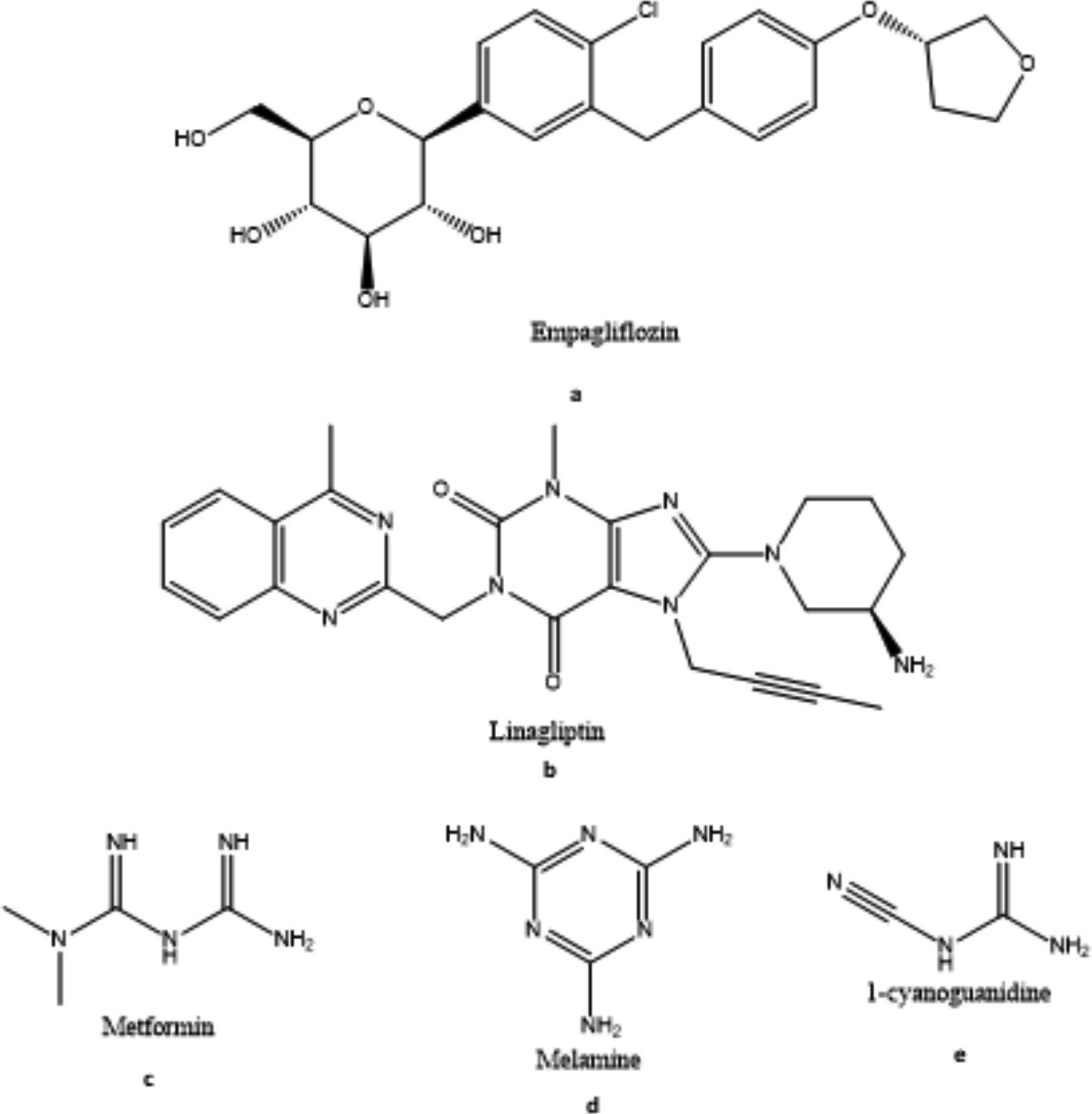
a. Chemical structure of Empagliflozin b. Chemical structure of Linagliptin c. Chemical structure of Metformin d. Chemical structure of Melamine e. Chemical structure of 1-cyanoguanidine

At epidemic levels, diabetes-related problems have become a worldwide problem [1]. Type 2 diabetes mellitus (T2DM) emergence, spread and the diversity of ambiguous physiological issues it generates are prompting necessity of presenting new pharmaceutical combinations that incorporate several pharmacological methods to improve glycemic control [2]. Considering this urgent necessity, a medicine with three components and effects must be introduced immediately. Hyperglycemic control medications like empagliflozin (EMP), shown in Fig. 1a, work by inhibiting sodium-glucose co-transporter II (SGLT2), a unique mechanism that may help to protect the heart and kidneys. They have a unique mode of action that is independent of pancreas cell function or insulin resistance. They might thus be used with other kinds of anti-diabetic drugs in addition to being used as a monotherapy [3–6]. These inhibitors filter and reabsorb glucose in the proximal tubules of the kidneys contributing to the maintenance of glucose homeostasis. This group’s additional advantages include a drop in systolic blood pressure and weight loss without the danger of decreasing blood glucose levels [5]. Linagliptin (LIN), Fig. 1b, is an antagonist of the dipeptidyl peptidase-4 (DPP-IV) enzyme, boosts glucose elimination by enhancing insulin production from pancreatic β-cells and inhibiting the enzymatic breakdown of two key hormones. This mechanism leads to increased glucose removal and insulin release [7]. The first-line treatment for hyperglycemia is metformin (MET), as indicated in Fig. 1c. it is an effective therapy for type 2 diabetes because it suppresses the liver’s glucose synthesis while simultaneously boosting insulin sensitivity in the targeted cell by restricting gastrointestinal absorption [8]. The detection and characterization of impurities in recently manufactured drugs has long been seen as one of the most essential aspects of drug development [9, 10]. That is because of the consequences of even slight quantities of impurities on pharmaceutical products’ efficacy, safety, and quality [11]. It is thus conceivable to measure EMP, LIN, and MET in a green way using the existing approach together with two of MET toxic pharmacopeial impurities [12] namely, melamine (MEL), Fig. 1d, and cyanoguanidine (CYG), Fig. 1e. In animal experiments, MEL has been found to induce renal inflammation, nephrolithiasis, and bladder cancer [13, 14]. Furthermore, since melamine has a positive charge, it is thought to bond with albumin. Arachidonic acid gets stuck to albumin. So, if melamine binds to albumin, it could take the place of arachidonic acid in the albumin-bound state. In the case of melamine exposure, this mechanism might be the source of inflammation-inducing mediators [15]. In the present study, molecular docking modeling is used to investigate this problem. CYG is also known to have an irritant impact on the skin and eyes [16]. AS far as we know, many HPLC methods have been published for the simultaneous measurement of EMP, LIN, and MET mixture alone or in combination with other anti-diabetic medications [17–23]. The FDA just approved a new combination tablet dosage form, and earlier published methods did not work with it well. After a series of hurdles in developing our current HPLC technique, including the ratio of the tablet, EMP: LIN: MET, 5: 1: 200 and the choice of ecologically acceptable solvents, we were ultimately able to establish a method that was more sensitive than the previously published HPLC methods. When we surmounted these obstacles, our approach excelled the previously published methods and became a significant method for the pharmaceutical industry. In a nutshell, the effort’s purpose was to design a procedure that is simple, sensitive, accurate, and time and cost efficient. According to the International Conference on Harmonization (ICH) standards [24], the present approach was completely validated and indicated no statistically significant changes when compared to a previously published method.

## Experimental

### Instruments

Waters Alliance 2690 HPLC separation unit, coupled with a quaternary pump, degasser, column compartment, auto-sampler, and Waters 996 photodiode array detector (DAD), was employed in the HPLC system (Milford, MA, USA) to apply the current technique. Empower 3 chromatography software was used for data processing and modification. Zorbax^®^ SB C_8_ analytical column (4.6 × 250 mm, 5 µm) served as the stationary phase. The pH readings were taken with a pH meter, model 3505 Jenway (Essex, UK). Soniclean 120T ultrasonic cleaner (Thebarton, South Australia, Australia) was utilized. Filtration of the mobile phase and sample was carried out using 0.45 mm MS^®^ Nylon membrane filters and 0.22 mm MS^®^ disposable syringe filters, respectively.

### Materials and reagents

#### Pure samples and pharmaceutical formulation

A generous donation of pharmaceutical-grade samples of EMP, LIN, and MET was made possible thanks to Eva Pharm Company for Pharmaceuticals and Chemicals (Al-Giza, Egypt), which provided samples with purities of 99.65%, 100.07%, and 99.84%, respectively. MEL and CYG were acquired from Sigma-Aldrich (St. Louis, MO, USA) with a purity of 99.9% for both of them. Boehringe Ingelheim Pharmaceuticals, Inc. (Ridgefield, CT, USA) and Eli Lilly Company (Indianapolis, IN, USA) made Trijardy XR^®^ tablet (batch no. 564785, labeled to contain 25 mg EMP, 5 mg LIN, and 1000 mg MET per tablet).

#### Chemicals and reagents

We employed HPLC-grade solvents, including acetonitrile and methanol from Fisher Scientific (Waltham, MA, USA) and Otsuka Pharmaceutical Co. (Cairo, Egypt) was the supplier of the double-distilled water. The ο-phosphoric acid and potassium dihydrogen phosphate were obtained from Sigma Aldrich (Saint Louis, MO, USA).

#### Standard solutions

In methanol, standard stock solutions of EMP, LIN, MET (1.0 mg/mL) were produced while MEL and CYG stock standard solutions were produced in distilled water. The EMP and LIN standard working solutions (100.0 µg/mL) were then prepared by diluting aliquots of the prepared standard stock solutions with the mobile phase.

#### Chromatographic conditions and optimization

**Fig. 2:**
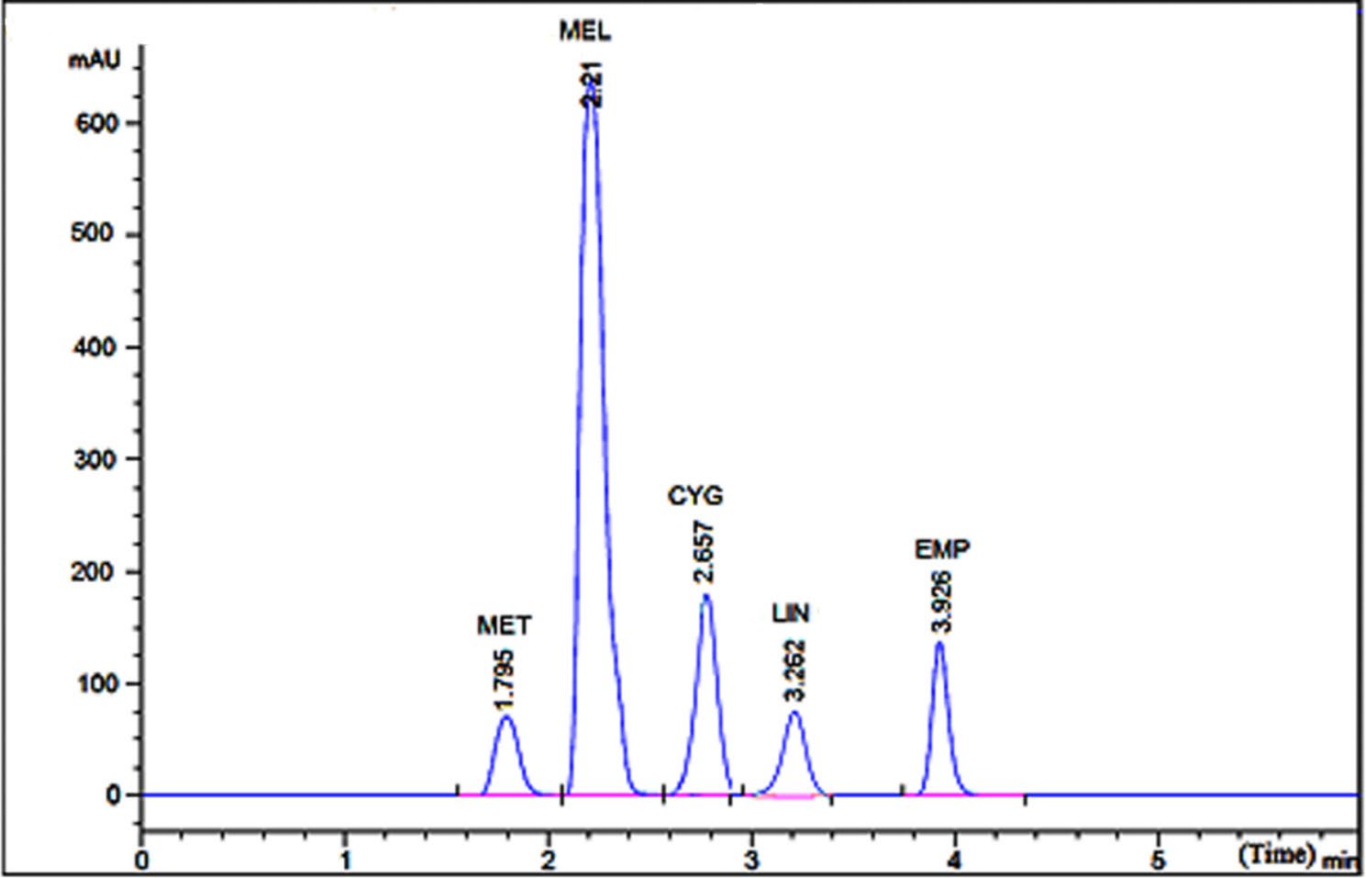
HPLC chromatogram for resolving EMP, LIN, and MET along with MEL and CYG using Zorbax^®^ SB C_8_ column (4.6 × 250 mm, 5 μm) and an isocratic mobile phase composed of acetonitrile: 0.05 M potassium di-hydrogen phosphate buffer treated with ο-phosphoric acid to get a pH of 4 (90: 10, v/v) with detection at 250 nm employing at a flow rate of 1.2 mL/min

Due to our intention of promoting an ecologically friendly approach, several harmful solvents were avoided and omitted from the studies. Various mobile phases containing water, acetonitrile, and methanol in various ratios were tested during the method development process, as well as water containing 0.1% formic acid and/or triethylamine in various ratios to acetonitrile and methanol, but all trials resulted in poor separation and/or resolution. At this stage, water was substituted with various ratios of potassium dihydrogen phosphate buffer with varying pH values and ratios and at this point, the analytes’ peaks were well separated but lacked adequate symmetrical peaks. In addition, the comparison between methanol and acetonitrile was done. Acetonitrile produced sharper symmetrical peaks and a higher sensitivity for the analytes in a short time. An ultrasonic degasser was employed to eliminate gas from the mobile phase, which was formed of 0.05 M potassium dihydrogen phosphate buffer (pH 4.0) and acetonitrile in the ratio of (10:90, v/v). Additional factors were studied, such as flow rate and column temperature, in order to achieve an efficient and satisfying separation. The drugs’ UV absorption spectra were recorded using a DAD detector, allowing the proper wavelength to be picked. Using a mobile phase of 0.05 M potassium di-hydrogen phosphate buffer (pH 4.0) and acetonitrile in the ratio of (10:90, v/v) in an isocratic elution program, the perfect separation was accomplished at a flow rate of 1.2 mL/min at 250 nm on a C_8_ column, where the separation was perfectly completed within a short time (less than 4 min.) (Fig. 2), specially for MET, MEL, and CYG due to their high polarities. The analysis was normally done after passing the mobile phase for half an hour, only to condition and pre-wash the stationary phase at a column temperature of 40°C. Each analyte was injected in triplicate after a 20.0 µL aliquot was filtered using 0.22 mm disposable syringe filters. Under these conditions, the retention times for EMP, LIN, MET, MEL, and CYG were 3.92, 3.26, 1.79, 2.21, and 2.65 min, respectively. Calibration curves were developed between peak areas and the concentration of each component, and linear regression parameters were then manipulated.

### Procedures

#### Linearity

Linearity was tested by serial dilution of each analyte from their corresponding stock or working standard solutions to obtain solutions with concentration ranges of 0.2–8.0 µg/mL for EMP, 0.3–9.0 µg/mL for LIN, 1.0–250.0 µg/mL for MET, and 1.0-100.0 µg/mL for MEL and CYG. The prepared solutions were injected in triplicates and chromatograms were generated under the stated optimized condition. The regression equations were constructed after calibration graphs were created, matching peak areas to the relevant concentrations.

### Assay of laboratory prepared mixtures

Calculated volumes of EMP and LIN were correctly transferred from their associated working solutions into a series of 10-mL volumetric flasks, while MET was transferred from its corresponding stock solution. Then, aliquots of MET’s official impurities, MEL and CYG, were mixed to produce different laboratory prepared mixtures. Volumes were refilled to the mark using the mobile phase, and the operation was then performed. Each component’s concentration was determined using its matching regression equation.

### Assay of the pharmaceutical tablet (trijardy XR^®^)

A mortar and pestle were used to grind ten Trijardy XR^®^ tablets into a fine powder. A quantity accurately weighed to include 25 mg EMP, 5 mg LIN, and 1000 mg MET was transferred to a 50-mL beaker, sonicated for 30.0 min with 25.0mL methanol, and then filtered into a 100-mL volumetric flask. Remaining methanol was used to wash the residue, which was then added to the filtrate solution. Methanol was used to get the final volume to the desired level. A tablet solution containing 2.5 µg/mL of EMP, 0.5 µg/mL of LIN, and 100.0 µg/mL of MET was prepared by adding 0.1 mL aliquot from the previously prepared solution to a 10-mL volumetric flask and diluting with mobile phase. To assess the concentrations of the three indicated drugs, chromatographic analysis was done as previously described.

## Results and discussion

Impurity profiling is becoming increasingly important for both new and old medication formulations, according to a variety of regulatory organizations [25]. The central issue during the establishment of that method was the separation and quantification of the stated medications, together with MEL and CYG, which are closely linked to MET (both structurally and chemically), in their combined tablet (25: 5: 1000, EMP: LIN: MET).

### Comparative study in terms of sensitivity and simplicity between the current method and other published methods

When compared to previously published techniques, the present methodology was confirmed to be the most sensitive since it could quantify the listed medicines at their lowest concentrations, as demonstrated in Table S1 (supplementary file). Aside from that, its detection and quantitation limits are much lower than those of other techniques. It was decided to employ the isocratic method of elution in this study rather than gradient elution, which had been previously used for these drugs. This made the technique more simpler than it had been before. In addition, the short separation time of 4 min and less solvents were used, resulting in both cost and time savings advantages. To sum up, our approach is the most precise, time-saving, and cost-effective when compared to the other published methods.

### Molecular docking simulation of melamine

Molecular docking was also used to demonstrate how MEL toxicity could be shown by its attachment to several of albumin’s known arachidonic acid binding sites. The results revealed the ability of MEL to bind at various different amino acid residues of albumin’s pocket either through hydrogen bonding or Vander wall force such as Gln221, Asn295, Asp451 and Arg222 with bond length ranging from 3.09 to 3.29 A^o^. Finally, regular and continuous interaction of MEL with albumin’s recognized arachidonic acid (ACA) may cause great injury to the health of individual, so it is very important to determine the studied drugs in presence of related toxic impurities as MEL to control the safety of the pharmaceutical product.

**Fig. 3:**
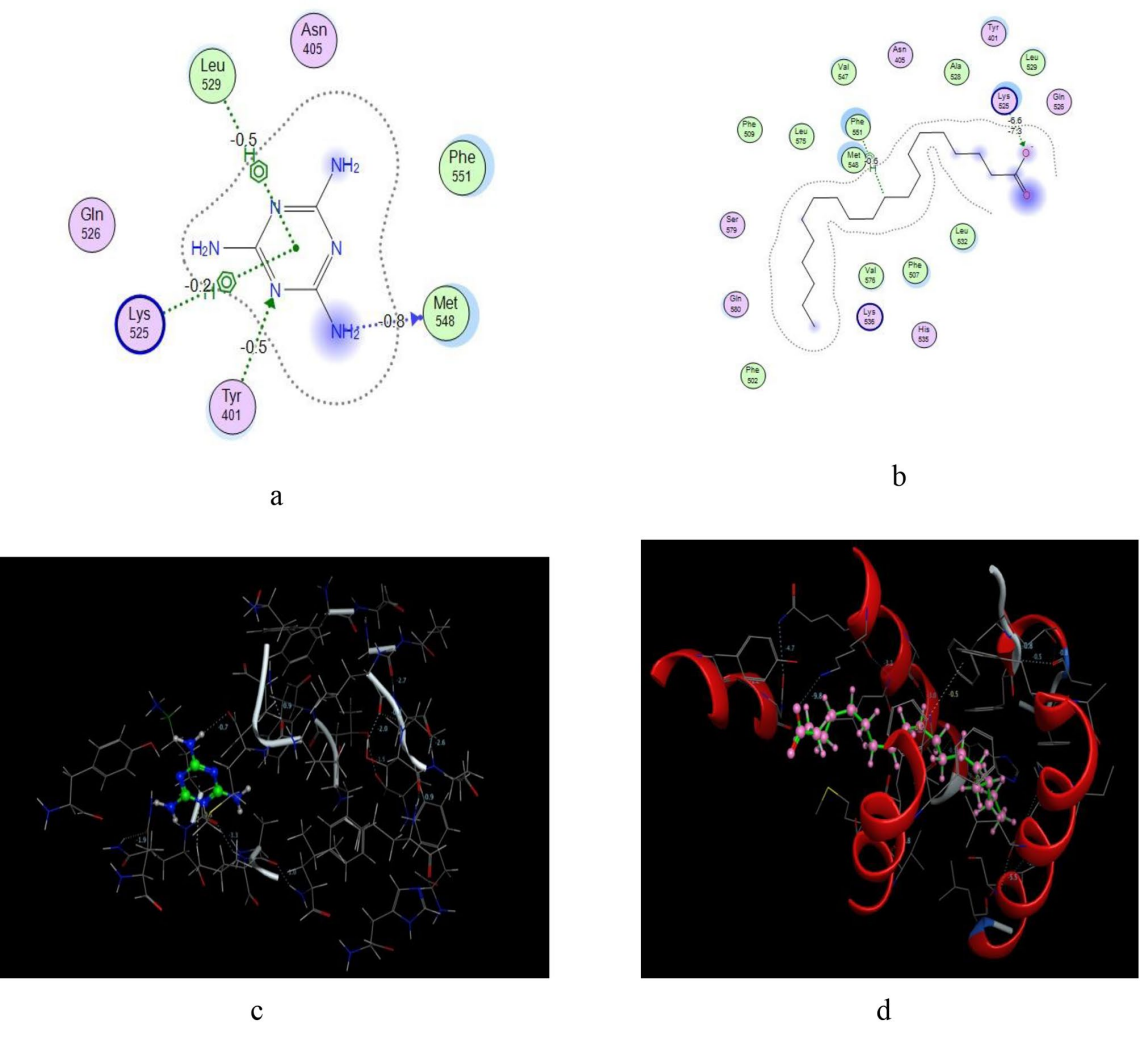
Molecular docking modeling of (a) melamine at arachidonic acid binding sites in albumin (b) arachidonic acid at albumin (c) 4 D plot of melamine at arachidonic acid binding sites in albumin (d) 4 D plot of arachidonic acid at albumin

Melamine has been shown to attach to several of albumin’s recognized arachidonic acid binding sites (Fig. 3**a, b, c and d)**. It is also observed that the bonding energy between melamine and albumin sites is negatively charged and that means no need of energy for that reaction (Fig. 3**a, b, c and d)**. Melamine binds at the same binding sites of arachidonic acid in albumin (Fig. 3**a and b)**. Figure 3**(c and d)** shows the 4-D plot for the binding of (a) melamine to albumin (b) arachidonic acid to albumin. More free arachidonic acid may be formed as a result of this. Melamine, on the other hand, does not bind to the extracellular signal-regulated kinase 2 (ERK2). As a result, melamine-induced inflammation cannot be caused via an ERK2-mediated signal transduction pathway. As a result, we believe that a higher amount of free arachidonic acid in the melamine-exposed condition may contribute more to inflammation.

### Assay of the combined tablet dosage form

EMP, LIN, and MET have been detected in Trijardy^®^ XR tablet using the current method, as shown in Table 1. The current method’s validity was further examined using a standard addition methodology. Obtaining the desired findings demonstrates that the proposed approach is suitable for regular analysis of the aforementioned medications in their combined pharmaceutical formulation without interference from excipients, as shown in Table 1.

**Table 1:** Results obtained by applying the current HPLC method for the determination of EMP, LIN, and MET along with MEL and CYG as MET potential official impurities in Trijardy XR^®^ tablets and application of standard addition technique

Pharmaceutical preparation								Current HPLC method				
								EMP		LIN		MET
**Trijardy** ^**®**^ **tablets**, **(25 mg EMP/5 mg LIN/1000 mg MET per tablet)**							**Mean ± SD**	101.20 ± 0.355		99.08 ± 1.272		99.59 ± 0.671
**Standard addition technique**												
**Current HPLC method**												
**Pharmaceutical formulation taken** **( µg/mL)**			**Pure drug added** **(µg/mL)**			**% Recovery** ^**a**^ **of EMP**			**% Recovery** ^**a**^ **of LIN**		**% Recovery** ^**a**^ **of MET**	
**EMP**	**LIN**	**MET**	**EMP**	**LIN**	**MET**							
2.5	0.5	100	2.00	0.40	80.00	101.02			99.82		99.72	
			2.50	0.50	100.00	100.47			101.61		101.11	
			3.00	0.60	120.00	99.76			100.52		101.58	
**Mean ± SD**						100.42 ± 0.630			99.08 ± 1.272		99.59 ± 0.671	

^a^ Mean of three determinations

MET, metformin; MEL, melamine; CYG, cyanoguanidine; LIN, linagliptin; EMP, empagliflozin

### Method validation

The present method has been thoroughly validated in accordance with the specifications of the ICH Q2 (R1) [24].

### Linearity

The linearity of an analytical procedure is its ability (within a given range) to obtain test results which are directly proportional to the concentration (amount) of analyte in the sample. The strong correlation coefficient (0.9999) of all the indicated drugs confirmed the good linearity of the current method. Table 2 summarizes the linearity ranges for various concentrations of EMP, LIN, MET, MEL, and CYG.

**Table 2:** Regression and validation parameters of the current HPLC method for the determination of EMP, LIN, and MET along with MEL and CYG as MET potential official impurities

Method parameter		Current HPLC method				
		EMP	LIN	MET	CYG	MEL
Linearity range		0.2–8.0 µg/mL	0.3–9.0 µg/mL	1.0–250.0 µg/mL	1.0–100.0 µg/mL	1.0–100.0 µg/mL
Slope		116.407	38.804	183.342	8.262	91.431
Intercept		-3.772	-1.964	-17.832	0.492	-14.033
Correlation coefficient (r)		0.9999	0.9999	0.9999	0.9999	0.9999
LOD^a^		0.05	0.09	0.19	0.2	0.1
LOQ^a^		0.15	0.27	0.58	0.9	0.8
Precision % RSD	Intra-day^b^	0.438	0.311	0.621	---	---
	Inter-day^c^	0.212	0.540	0.275	---	---

^a^ LOD and LOQ are calculated according to ICH, 3.3 × SD of the intercept/slope and 10 × SD of the intercept /slope, respectively

^b^Intra-day precision [average of three different concentrations of three replicates each (n = 9) within the same day]

^c^Inter-day precision [average of three different concentration of three replicates each (n = 9) repeated on 3 successive days

### Detection and quantitation limits

The limit of detection (LOD) and the limit of quantitation (LOQ) were calculated according to

ICH guidelines from the following equations:

LOD = 3.3 σ / S

LOQ = 10 σ / S

Where σ is the standard deviation of y-intercepts of regression lines and S is the slope of the calibration curve. Table 2 displays the values for the aforementioned five components.

### Accuracy

Five different concentrations of each component were prepared along their corresponding linearity ranges and analysed in triplicate by the proposed method. The concentrations were then anticipated from the corresponding regression equations. Good mean percentage recoveries for all the components reveal the accuracy of the method. In triplicate, five concentration levels of EMP (0.3, 0.5, 2.0, 5.0, and 7.0 µg/mL), LIN (0.5, 2.0, 4.0, 5.0, and 7.0 µg/mL), and EMP (4.0, 8.0, 15.0, 30.0, and 150.0 µg/mL) were analyzed. Their found concentrations and average percentage recoveries (R%) were then computed. **Table S2 (supplementary file)** summarizes the results and represents the accuracy of the existing procedure.

### Precision

The drug components were prepared at three different concentration levels. Each concentration is then analyzed three times either intra-daily for investigating “repeatability”, which expresses the precision under the same operating conditions over a short interval of time or inter-daily for evaluating “intermediate precision”, which expresses within-laboratories variations: different days, different analysts, different equipment.

Precision was investigated by evaluating EMP (0.5, 2.0, and 5.0 µg/mL), LIN (2.0, 4.0, and 5.0 µg/mL), and MET (8.0, 15.0, and 30.0 µg/mL) using the current method. Table 2 shows that satisfactory values of intra-day and inter-day relative standard deviation (RSD%) represented the suggested methods’ strong repeatability and intermediate precision.

### Selectivity and specificity

The present method’s selectivity and specificity were proven by analyzing laboratory-prepared mixtures of EMP, LIN, MET, MEL, and CYG at various concentration ratios within the examined linearity ranges. Each synthetic mixture was supplemented with MEL and CYG at concentrations ranging from 5.0 to 50.0% of the MET concentration. Each combination was determined in triplicate, and the mean recovery and standard deviation for each compound were computed and included in Table 3. The suggested techniques’ excellent selectivity was shown by low SD values (less than 2.0), indicating their capacity to resolve and quantify the five analytes in different concentration ratios. The effective isolation of the three anti-diabetic drugs from MET impurities (MEL and CYG) confirmed the specificity of the proposed approach. The specificity was determined using a photodiode array detector, which confirmed the purity of peaks of all analytes with no co-elution of any of the related substances with the three drug peaks, as well as no co-elution of any of the added inactive ingredients in combined dosage form with the drug peaks. A further demonstration of the high specificity and applicability of the current method to the assay of the three anti-diabetic drugs in their triple combination tablets without any interfering effect from excipients present in tablet extract is provided in Table 1, which shows adequate recovery values with low values of SD not exceeding 2.0 and no interfering effect from excipients present in tablet extract.

**Table 3:** Laboratory prepared mixture results of the current HPLC method for the determination of EMP, LIN, and MET along with MEL and CYG as MET potential official impurities

MIX	EMP	EMP Found	% Recovery ^a^ of EMP	LIN	LIN Found	% Recovery ^a^ of LIN	MET	MET Found	% Recovery ^a^ of MET	MEL	CYG
1	5.00	5.01	100.20	5.00	4.92	98.58	5.00	4.94	98.95	2.50	2.50
2^b^	5.00	5.01	100.20	1.00	1.01	101.01	200.00	203.17	101.58	10.00	10.00
3	2.50	2.53	101.21	0.50	0.49	98.94	100.00	100.45	100.45	20.00	20.00
4	4.00	3.96	99.07	3.00	2.96	98.76	10.00	9.86	98.61	4.00	4.00
5	7.00	6.90	98.59	6.00	6.03	100.58	20.00	20.01	100.05	5.00	5.00
	Mean ± SD		99.83 ± 1.022	Mean ± SD		99.58 ± 1.131	Mean ± SD		99.93 ± 1.196		

^a^ Mean of three determinations

^b^ Ratio present in tablet dosage form

EMP, empagliflzin; LIN, linagliptin; MET, metformin

### Robustness

Changing the pH of the buffer (± 0.1) and the mobile phase ratio (± 1.0 mL) allowed us to reevaluate the present procedure. **Table S3 (supplementary file)** shows that the system suitability parameters have not changed significantly and acceptable RSD % values have been developed.

### System suitability parameters

After optimizing the various system suitability parameters, the performance of the proposed HPLC technique was effectively assured [26]. The obtained values for retention time, capacity factor, resolution, and tailing factors of EMP, LIN, MET, MEL, and CYG were in excellent agreement with USP reference values [27], as shown in Table 4.

**Table 4:** System suitability parameters of the current HPLC method for the determination of EMP, LIN, and MET along with MEL and CYG as MET potential official impurities

Parameter	MET		MEL		CYG		LIN		EMP	Reference value [27]
Selectivity(α)^c^		1.23		1.19		1.23		1.20		≥ 1
Resolution(R_s_)^d^		2.14		3.24		3.32		5.34		> 2
Capacity factor (K’)	1.02		2.34		6.54		7.43		8.32	1–10
Tailing factor(T)	1.12		1.22		1.18		1.17		1.18	≤ 2
Column efficiency (N)	2165		2534		3455		6543		7544	The higher the value, the more efficient the column is
Height equivalent to theoretical plate(cm/plate)	0.01154		0.00986		0.00723		0.00382		0.00331	The smaller the value, the higher the column efficiency
Retention time (R_t_, min)	1.79		2.21		2.65		3.26		3.92	---

MET, metformin; MEL, melamine; CYG, cyanoguanidine; LIN, linagliptin; EMP, empagliflozin

### Statistical analysis

A statistical comparison was made between the findings produced by the present HPLC technique and those obtained by a previously described method [21]. The estimated t and F values are less than the tabular values, indicating that there is no statistically significant difference between them as illustrated in Table 5.

**Table 5:** Statistical comparison of the results obtained from EMP, LIN, and MET in Trijardy® tablet by the Current HPLC method and a reported HPLC method

Parameters	EMP		LIN		MET	
	Reported method ^a^ [23]	Current method	Reported method ^a^ [23]	Current method	Reported method ^a^ [23]	Current method
Mean	99.65	101.20	99.65	99.08	99.96	99.59
SD	0.388	0.355	0.357	1.272	0.250	0.671
Variance	0.151	0.126	0.127	1.618	0.063	0.450
t-test^b^	---	0.20 (2.78)	---	1.43 (2.78)	---	1.12 (2.78)
F-test^b^	---	1.19 (19.00)	---	12.70 (19.00)	---	7.20 (19.00)

^a^ Reversed phase HPLC-DAD employing Agilent C18 column (4.6 × 250 mm, 5 μm p.s.) and a mobile phase composed of methanol and 0.01 M sodium di-hydrogen orthophosphate buffer of pH 2.55 (adjusted with ortho-phosphoric acid) eluted in a gradient mode with detection at 218 and 224 nm.

^b^ The theoretical values of t and F at P = 0.05 are (2.78) and (19.00), respectively where n = 3.

EMP, empagliflzin; LIN, linagliptin; MET, metformin.

## Conclusion

The current research is regarded as the most sensitive, selective, and trustworthy HPLC-DAD for the simultaneous quantification of EMP, LIN, and MET with MET related potentially pharmacopeial impurities MEL and CYG. The current method has an advantage over all previously published methods in that it allows for the neat and efficient separation of the three anti-diabetic medicines from MET pharmacopeial impurities. As a result, it might be regarded as basic stability-indicating assay and applied to the actual tablet (not a synthetic one). Finally, the present method’s dependability was shown by its validation in accordance with ICH criteria and its effective use for the analysis of three anti-diabetic medications in tablet dosage form without interference from inactive substances. For regular examination of the mentioned drugs in their pure form or their newly approved triple combination tablet, it provides easy, cost-effective, and stability-indicating alternatives to previously described chromatographic procedures.

## Electronic supplementary material

Below is the link to the electronic supplementary material.

Supplementary Material 1

## Data Availability

All data generated or analyzed during this study are included in this published article.

## Abbreviations

CYG: Cyanoguanidine
DAD: Diode Array Detector;
DPP-IV: Dipeptidyl Deptidase-4
EMP: Empagliflozin
ICH: International Conference on Harmonization
LIN: Linagliptin
LOD: Limit of detection
LOD: Limit of quantitation
MET: Metformin
MEL: Melamine
SGLT2: Sodium-glucose co-transporter II
T2DM: Type 2 diabetes mellitus
USP: United States Pharmacopeia
