# Supplementary material for: HPLC-DAD technique for the quantification of a recently approved anti-diabetic triple combination along with two toxic official impurities: Toxicity confirmation aided by molecular docking application
Source: BMC Chem. 2023 Mar 15;17(1):18. doi: 10.1186/s13065-023-00927-0 (PMC10018879; doi:10.1186/s13065-023-00927-0)
Supplement: Supplementary file 1 — Supplementary Material 1 [file 13065_2023_927_MOESM1_ESM.docx]

| **Application** | **Total HPLC run time for the cited drugs** | **Elution mode** | **LOQ (μg/mL)** | | | **LOD (μg/mL)** | | | **Linearity range (μg/mL)** | | | **Parameters** |
| --- | --- | --- | --- | --- | --- | --- | --- | --- | --- | --- | --- | --- |
|  |  |  | **MET** | **LIN** | **EMP** | **MET** | **LIN** | **EMP** | **MET** | **LIN** | **EMP** |  |
| Real Trijardy® tablet  Impurity profiling  Green analysis | 3.92 | Isocratic | 0.71 | 0.27 | 0.15 | 0.23 | 0.09 | 0.05 | 1.0-250.0 | 0.3-9.0 | 0.2-8.0 | **Current HPLC method^a^** |
| Laboratory synthetic tablet not a real tablet  Impurity profiling  Green analysis | 8.53 | Gradient | 8.93 | 3 7.62 | 27.52 | 2.68 | 2.29 | 8.26 | 15–300 | 12.5–200 | 30–400 | **Reported HPLC method A [23]** |
| Single tablets from different companies  not combined dosage form | 5.99 | Gradient | 174.32 | 3.43 | 5.32 | 57.32 | 1.10 | 1.32 | 500.0–3500.0 | 10.0-70.0 | 20.0-140.0 | **Reported HPLC method B [17]** |
| Quality by design no tablet or other applications | 8.42 | Gradient | 2.98 | 26.87 | 16.05 | 0.98 | 8.77 | 5.29 | 10.0-70.0 | 10.0-70.0 | 30.0-210.0 | **Reported HPLC method C [20]** |

**Table** **S1** : Comparison between the current HPLC method for the determination of EMP, LIN, and MET along with MEL and CYG as MET potential official impurities and three other published HPLC methods in terms of sensitivity and simplicity.

^a^ The most sensitive and simplest method

EMP, empagliflzin; LIN, linagliptin; MET, metformin

| **EMP** | **EMP found** | **% Recovery ^a^ of EMP** | **LIN** | **LIN found** | **% Recovery ^a^ of LIN** | **MET** | **MET found** | **% Recovery ^a^ of MET** |
| --- | --- | --- | --- | --- | --- | --- | --- | --- |
| 0.30 | 0.30 | 100.23 | 0.50 | 0.49 | 99.50 | 4.00 | 4.03 | 100.76 |
| 0.50 | 0.50 | 101.56 | 2.00 | 1.99 | 99.58 | 8.00 | 8.17 | 102.19 |
| 2.00 | 1.96 | 98.40 | 4.00 | 3.93 | 98.32 | 15.00 | 14.98 | 99.92 |
| 5.00 | 4.95 | 99.13 | 5.00 | 4.92 | 98.42 | 30.00 | 29.66 | 98.87 |
| 7.00 | 6.92 | 98.95 | 7.00 | 7.06 | 100.94 | 150.00 | 150.63 | 100.42 |
| **Mean ± SD** | | 99.56±1.257 | **Mean ± SD** | | 99.35±1.056 | **Mean ± SD** | | 100.43±1.217 |

**Table S2:** Accuracy results of the current HPLC method for the determination of EMP, LIN, and MET along with MEL and CYG as MET potential official impurities.

^a^ Mean of three determinations

EMP, empagliflzin; LIN, linagliptin; MET, metformin.

**Table S3 :** Results ^a^ of robustness testing of the current HPLC method for the determination of EMP, LIN, and MET along with MEL and CYG as MET potential official impurities.

| **Condition** | **Drug** | **R_t_** | **N** | **T** | **R_S_** |
| --- | --- | --- | --- | --- | --- |
| **Change mobile phase composition**  **acetonitrile: 0.05 M phosphate**  **buffer (91:9 v/v), (90:10, v/v)** | **MET** | 0.456 | 1.344 | 0.546 | 1.123  1.234  0.765  0.743 |
|  | **MEL** | 0.467 | 0.675 | 0.975 |  |
|  | **CYG** | 1.766 | 0.986 | 1.546 |  |
|  | **LIN** | 0.976 | 0.754 | 0.542 |  |
|  | **EMP** | 0.876 | 0.459 | 0.832 |  |
| **Change pH of buffer**  **pH (4.0±0.1;4.1 and 3.9)** | **MET** | 0.654 | 0.560 | 1.241 | 0.676  1.543  0.655  1.534 |
|  | **MEL** | 0.654 | 1.854 | 1.356 |  |
|  | **CYG** | 0.457 | 1.534 | 1.656 |  |
|  | **LIN** | 0.986 | 1.256 | 1.876 |  |
|  | **EMP** | 1.543 | 1.745 | 1.345 |  |

^a^ % RSD values

R_t_, retention time; N, number of theoretical plates; T, tailing factor; R_s_, resolution.

MET, metformin; MEL, melamine; CYG, cyanoguanidine; LIN, linagliptin; EMP, empagliflozin.
